# Vitamin D Promotes Skeletal Muscle Regeneration and Mitochondrial Health

**DOI:** 10.3389/fphys.2021.660498

**Published:** 2021-04-14

**Authors:** Christine M. Latham, Camille R. Brightwell, Alexander R. Keeble, Brooke D. Munson, Nicholas T. Thomas, Alyaa M. Zagzoog, Christopher S. Fry, Jean L. Fry

**Affiliations:** ^1^Department of Athletic Training and Clinical Nutrition, University of Kentucky, Lexington, KY, United States; ^2^Center for Muscle Biology, College of Health Sciences, University of Kentucky, Lexington, KY, United States

**Keywords:** vitamin D receptor, vitamin D, 25(OH)D, calcitriol, skeletal muscle injury, satellite cells, reactive oxygen species, skeletal muscle regeneration

## Abstract

Vitamin D is an essential nutrient for the maintenance of skeletal muscle and bone health. The vitamin D receptor (VDR) is present in muscle, as is CYP27B1, the enzyme that hydroxylates 25(OH)D to its active form, 1,25(OH)D. Furthermore, mounting evidence suggests that vitamin D may play an important role during muscle damage and regeneration. Muscle damage is characterized by compromised muscle fiber architecture, disruption of contractile protein integrity, and mitochondrial dysfunction. Muscle regeneration is a complex process that involves restoration of mitochondrial function and activation of satellite cells (SC), the resident skeletal muscle stem cells. VDR expression is strongly upregulated following injury, particularly in central nuclei and SCs in animal models of muscle injury. Mechanistic studies provide some insight into the possible role of vitamin D activity in injured muscle. *In vitro* and *in vivo* rodent studies show that vitamin D mitigates reactive oxygen species (ROS) production, augments antioxidant capacity, and prevents oxidative stress, a common antagonist in muscle damage. Additionally, VDR knockdown results in decreased mitochondrial oxidative capacity and ATP production, suggesting that vitamin D is crucial for mitochondrial oxidative phosphorylation capacity; an important driver of muscle regeneration. Vitamin D regulation of mitochondrial health may also have implications for SC activity and self-renewal capacity, which could further affect muscle regeneration. However, the optimal timing, form and dose of vitamin D, as well as the mechanism by which vitamin D contributes to maintenance and restoration of muscle strength following injury, have not been determined. More research is needed to determine mechanistic action of 1,25(OH)D on mitochondria and SCs, as well as how this action manifests following muscle injury *in vivo*. Moreover, standardization in vitamin D sufficiency cut-points, time-course study of the efficacy of vitamin D administration, and comparison of multiple analogs of vitamin D are necessary to elucidate the potential of vitamin D as a significant contributor to muscle regeneration following injury. Here we will review the contribution of vitamin D to skeletal muscle regeneration following injury.

## Introduction

Vitamin D is a fat-soluble vitamin with critical roles for bone and skeletal muscle health. Dietary sources of vitamin D include fortified foods, such as bread and milk, fatty fish, some mushrooms, and dietary supplements. Additionally, upon exposure to ultraviolet B rays, human skin can convert 7-dehydrocholesterol to vitamin D. After ingestion or synthesis, vitamin D is activated by two hydroxylation reactions, occurring mainly in the liver and kidneys, to form biologically active 1,25-hydroxyvitamin D [1,25(OH)D]. In the liver, vitamin D-25-hydroxylase converts vitamin D to 25(OH)D. The second hydroxylation is carried about by CYP27B1-encoded 1α-hydroxylase (CYP27B1), which yields 1,25(OH)D (Bikle, 2000). Though most 1α-hydroxylase activity occurs in the kidneys, the enzyme is expressed in other cell types including macrophages, monocytes, and muscle fibers (Pojednic and Ceglia, 2014). Bioactive 1,25(OH)D is a transcription factor affecting the expression of hundreds of genes (Bikle, 2000). In this capacity, 1,25(OH)D first binds to its nuclear receptor, the vitamin D receptor (VDR). This complex binds to a retinoid X receptor (RXR) to form a VDR-RXR heterodimer. The heterodimer interacts with genomic vitamin D response elements (VDREs) to regulate transcription (Bikle, 2000).

Since serum 25(OH)D best reflects vitamin D exposure and absorption, and has a relatively long half-life, this metabolite is used to evaluate vitamin D status (Holick, 2009). Serum 25(OH)D < 30 nmol/L (12 ng/ml) is defined as vitamin D deficiency while 25(OH)D concentrations between 30 and 50 nmol/L (12–20 ng/ml) are categorized as vitamin D insufficiency (Ross, 2011). To support bone health, 25(OH)D concentrations > 50 nmol/L (20 ng/ml) are considered adequate (Houston et al., 2007). However, evidence indicates that full suppression of parathyroid hormone, which is released when vitamin D is needed, occurs at a concentration of about 100 nmol/L (40 ng/ml; Ginde et al., 2012), suggesting that optimal vitamin D status may be above cut-points established to prevent vitamin D deficiency symptoms. These discrepancies in estimates of sufficiency are reflected by a lack of consensus on vitamin D cut points in skeletal muscle literature (Table 1), making comparisons between studies difficult.

**Table 1:** 25-hydroxyvitamin D [25(OHD)] cut points vary significantly across skeletal muscle studies.

	25(OH)D cut points in human vitamin D/skeletal muscle studies			
	Deficient	Insufficient	Sufficient	Optimal
National Academies/Institutes of Medicine standards	<30 nmol/L	30 to <50 nmol/L	≥50 nmol/L	N/A
Ranathunga et al., 2019	<25 nmol/L	25 to <50 nmol/L	N/A	N/A
Bang et al., 2018	<50 nmol/L	50 to <100 nmol/L	≥100 nmol/L	N/A
Dzik et al., 2018	30 to <50 nmol/L	N/A	≥50 nmol/L	N/A
Jang et al., 2020	<25 nmol/L	25 to <50 nmol/L	50 to <75 nmol/L	≥75 nmol/L
Kitsu et al., 2020	<50 nmol/L	50 to >75 nmol/L	>75 nmol/L	N/A
Montenegro et al., 2020	N/A	N/A	50 to 100 nmol/L	>100 nmol/L

Since sunlight exposure often contributes an appreciable amount to overall vitamin D supply, those living in latitudes above 43°N are more likely to be vitamin D deficient (Ross, 2011). For example, participants living in Erie, Pennsylvania were more likely to be vitamin D deficient compared with those in Bradenton, Florida (Leary et al., 2017). People with deeper skin tones and higher body fat are also more likely to be deficient (Leary et al., 2017). A classical 1,25(OH)D function is to promote intestinal calcium absorption to maintain blood calcium and bone mineralization (Ross, 2011). Severe vitamin D deficiency causes rickets in children and osteomalacia in adults, which is characterized by a softening and weakening of the bones (Nair and Maseeh, 2012). Vitamin D insufficiency is associated with inadequate bone health leading to loss of bone density, fractures, muscle weakness, osteopenia, and osteoporosis (Holick and Gordon, 2011).

Vitamin D status is also associated with muscle strength outcomes across a broad range of age groups (Redzic et al., 2013). In older adults, plasma 25(OH)D concentrations < 25 nmol/L are associated with significantly lower grip strength (Ranathunga et al., 2019). In a study of younger adults, including participants with deficient and optimal vitamin D status, higher baseline 25(OH)D concentrations predicted the restoration of strength following an intense resistance exercise bout (Barker et al., 2013). Though vitamin D supplementation studies have methodological differences in dosing, length of intervention, and participant characteristics (including baseline vitamin D status), a wealth of data indicates that correcting vitamin D status through supplementation improves muscle strength. A 2015 meta-analysis including primarily younger participants with 25(OH)D concentrations < 25 nmol/L showed vitamin D supplementation ranging from 4,000 to 60,000 IU per week significantly improved both upper and lower body strength (Tomlinson et al., 2015). An in-depth review of vitamin D supplementation and skeletal muscle strength outcomes in human participants may be found elsewhere (Chiang et al., 2017). Here we will review the contribution of vitamin D to skeletal muscle regeneration following injury.

## Skeletal Muscle Damage and Regeneration Throughout the Lifespan

**Figure 1:**
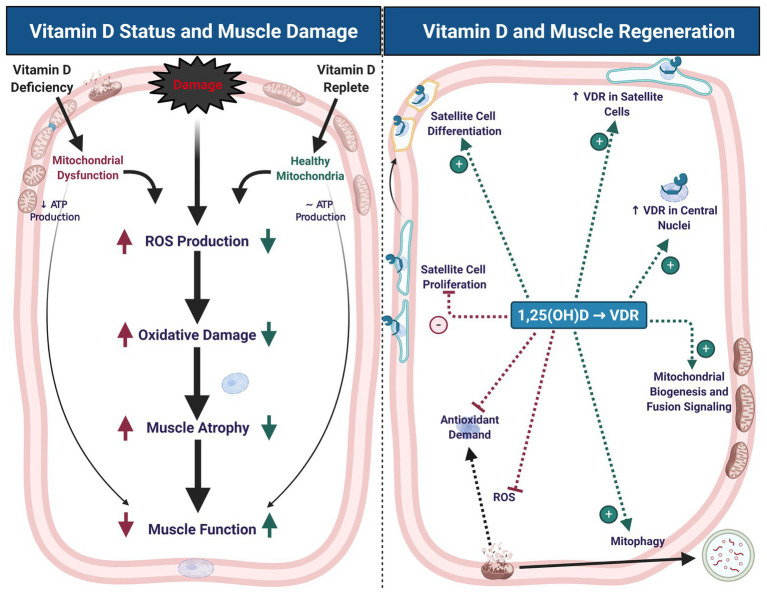
Vitamin D status contributes to muscle damage and regeneration. Vitamin D deficiency leads to mitochondrial dysfunction, decreased adenosine triphosphate (ATP) production, increased reactive oxygen species (ROS) production, oxidative damage, muscle atrophy, and impaired muscle function. These symptoms of deficiency may exacerbate similar symptoms that typically occur with muscle damage (left panel). During muscle regeneration (right panel), hydroxylated, activated vitamin D [1,25(OH)D] stimulates an increase in vitamin D receptor (VDR) abundance in satellite cells and central myonuclei. These changes in VDR abundance are accompanied by inhibition of satellite cell proliferation and stimulation of differentiation, which may contribute to maintenance of satellite cell self-renewal capacity. Signaling at the VDR also increases mitochondrial biogenesis and fusion signaling, inhibit ROS production, and thereby mitigate antioxidant demand, which may contribute to a more competent regenerative phenotype. Created with BioRender.com

Skeletal muscle is a remarkably plastic tissue, capable of robust adaptation and regeneration in response to stress and damage (Figure 1). Muscle damage can occur as a result of crush injury, ischemia-reperfusion injury, and resistance exercise, among other stimuli (McGeachie and Grounds, 1987; Roth et al., 1999, 2000; Mackey and Kjaer, 2017; Kuroda et al., 2020) and pre-clinical injury models in rodents offer reproducible and controlled experimental models (Hardy et al., 2016). The best-studied model of muscle damage in humans is unaccustomed resistance exercise, with high load eccentric muscle contractions being a significant driver of muscle damage (Roth et al., 1999, 2000; Damas et al., 2016; Mackey and Kjaer, 2017).

Severe muscle damage is characterized by disruption of muscle fiber integrity leading to impairments in structure and function of damaged muscle (Roth et al., 1999, 2000; Mackey and Kjaer, 2017). Muscle fiber damage is identified by Z-disc streaming and a smeared appearance of sarcomeres, indicating ultrastructural damage (Lieber and Fridén, 1999; Damas et al., 2016). This damage is further defined by severe disruption of the arrangement and structure of contractile proteins (Fridén et al., 1983a,b; Brown et al., 1997; Roth et al., 1999, 2000; Koh and McNally, 2007; Damas et al., 2016), leaving behind necrotic zones where regeneration of the muscle fiber can be initiated (Mackey and Kjaer, 2017). Necrotic zones in damaged muscle fibers serve as loci for neutrophil and macrophage accumulation (Mackey and Kjaer, 2017), a common theme of cellular damage (Uderhardt et al., 2019). In these damaged areas, cell proliferation is increased in response to heightened apoptosis and muscle fiber biogenesis, and this increased cellular turnover aids in the regeneration of viable muscle tissue (Mackey and Kjaer, 2017).

Muscle fiber damage is a unique form of cellular stress whereby the basement membrane of the damaged fiber is preserved, allowing for regeneration and adaptive remodeling of the fiber, as opposed to *de novo* fiber formation (Fridén et al., 1983a,b; Mackey and Kjaer, 2017). This regenerative potential of muscle fiber damage is unique, as comparable cellular stress in mitosis-competent cell types often necessitates *de novo* cell formation (Liao et al., 1997; Griffiths et al., 1999). The unique nature of muscle fiber damage and robust regenerative potential is owed to its resident stem cell population and multinucleation (Lepper et al., 2011). Skeletal muscle stem cells are termed satellite cells (SC), due to their residency on the periphery of the muscle fiber, between the basal lamina and sarcolemma (Katz, 1961; Mauro, 1961). SCs are indispensable for regeneration after severe muscle injury (McCarthy et al., 2011; Murphy et al., 2011; Sambasivan et al., 2011; Lepper et al., 2011), and are characterized by constitutive expression of Pax7 (Seale et al., 2000). SCs are activated after damage to the surrounding muscle and undergo asymmetric division to generate a “sister” and a “daughter” cell. The sister cell is able to return to quiescence, maintaining the resident SC pool. The daughter cell differentiates, progresses through the myogenic program, and eventually fuses with the surrounding muscle tissue and donates its nucleus (Zammit et al., 2004).

**Figure 2:**
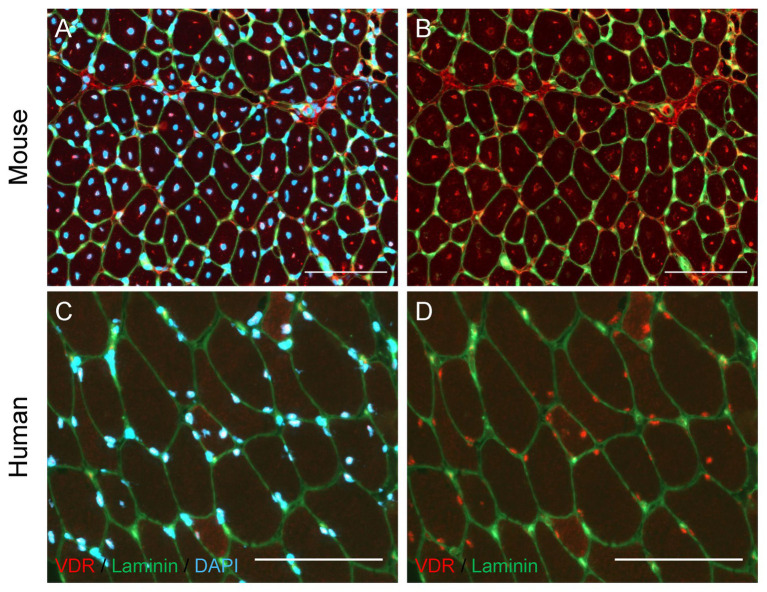
Localization of Vitamin D Receptor (VDR) in regenerating mouse and human skeletal muscle. **(A,B)** Muscle regeneration was induced in the mouse tibialis anterior muscle by injecting BaCl_2_ (1.2%), and the tibialis anterior muscle was harvested 7 days following injury. Immunohistochemical analysis revealed regenerating fibers with centrally-located myonuclei (A, DAPI) show strong positive VDR expression (B, red puncta) in myonuclei. **(C,D)** A vastus lateralis biopsy was obtained from a human research participant 7 days after severe thermal injury, which we have shown to induce significant skeletal muscle regeneration (Fry et al. 2016). Myonuclear localization of VDR (D, red puncta) is observed, with the strongest VDR intensity present within myonuclei (C, DAPI) of small, regenerating fibers. In all images, laminin (green) denotes the fiber border. In images A and C, DAPI (blue) denotes nuclei. In images B and D, DAPI is omitted to allow visualization of VDR staining within nuclei. Scale bar = 100 μm.

Recent evidence suggests that vitamin D signaling also contributes to muscle regeneration. In skeletal muscle of mature and aged mice, protein expression of VDR is closely associated with serum concentration of 25(OH)D (Srikuea et al., 2020). The VDR and vitamin D-activating enzyme CYP27B1 are lowly expressed in homeostatic skeletal muscle *in vitro* and *in vivo*, evidenced by immunocytochemical and immunohistochemical visualization and immunoblotting in both C2C12 myoblasts and whole mouse muscle (Srikuea et al., 2012, 2020). After muscle injury, the minimal expression of VDR and CYP27B1 observed in homeostatic conditions is starkly augmented. Vitamin D receptor expression is marginally detectable in uninjured skeletal muscle but is highly expressed and localized to regenerating muscle fibers after muscular injury (Figure 2; Srikuea et al., 2012), and is colocalized with central myonuclei (Srikuea and Hirunsai, 2016). Furthermore, endogenous VDR is expressed at detectable levels in SCs in regenerating skeletal muscle after injury, substantiated by colocalization of the Pax7 transcription factor and VDR protein (Srikuea and Hirunsai, 2016). The role of the vitamin D system in muscle regeneration is further supported by rapidly increased Pax7 and VDR protein expression in skeletal muscle initiating a repair response after an acute bout of damaging high-intensity exercise (Puangthong et al., 2020), demonstrating that the myogenic repair system and vitamin D system are both rapidly and concurrently initiated after damage to skeletal muscle.

Although muscle regeneration is highly efficient, the regenerative process can be compromised in pathological conditions (myopathies) and in aged individuals. Compromised regenerative processes in skeletal muscle during aging provide a particularly interesting area of study, as they are multifactorial and complex. Skeletal muscle mass peaks around the third decade of life and gradually decreases over time, with observable loss starting in the fifth decade (Janssen et al., 2000). A hallmark of age-related muscle atrophy is an imbalance between muscle protein synthesis and degradation. During aging, research has shown increases in protein degradation signaling (Cai et al., 2004) and anabolic resistance (Welle et al., 1993), which contribute to the observed loss of muscle mass. Additionally, impaired mitochondrial function and resultant excessive ROS production have been implicated in age-related muscle loss (Short et al., 2005). These declines in mitochondrial health have been attributed to impaired mitochondrial processes such as fusion and fission (Chabi et al., 2008; Gouspillou et al., 2014; Leduc-Gaudet et al., 2015; Liu et al., 2020), mitophagy (García-Prat et al., 2016), and biogenesis (Chabi et al., 2008). Further, the loss in skeletal muscle mass is coupled with decreases in the resident SC pool (Shefer et al., 2006) and SC activity (Conboy, 2003). Age-associated reductions in abundance and functionality of SCs induce significant decrements in the muscles’ regenerative capacity, which is often associated with accumulation of fibrotic material. This increase in fibrosis may be due to SC transition to a fibrogenic phenotype (Stearns-Reider et al., 2017) and resultant decline in SC-mediated regulation of fibroblast activity (Fry et al., 2017).

In mice, VDR protein expression is increasingly elevated from development through maturation and aging (Srikuea et al., 2020); however, a study in human muscle showed an inverse relationship between skeletal muscle VDR expression and age (Bischoff-Ferrari et al., 2004). VDR expression is strongly associated with changes in circulating 25(OH)D concentrations following vitamin D_3_ supplementation in older women with vitamin D insufficiency (25OHD of 46.3 ± 9.5 nmol/L; Ceglia et al., 2013). In mice, elevated VDR expression in aged skeletal muscle is also associated with central nucleation of muscle fibers (Srikuea et al., 2020) – typically indicative of muscle fibers recovering from a damaging stimulus. Further understanding the environment facilitating deficits in muscle regeneration, and perhaps the contribution of vitamin D signaling, could lead to the development of novel therapeutic strategies that support greater myogenic potential throughout the lifespan.

## Vitamin D and Mitochondrial Health

Vitamin D deficiency and insufficiency, when combined typically defined as 25(OH)D concentrations < 50 nmol/L, are associated with muscle atrophy and deficits in muscle strength in several clinical models (Sato et al., 2005; Tagliafico et al., 2010; van Langenberg et al., 2014; Almurdhi et al., 2017; Bang et al., 2018). While many studies of vitamin D deficiency and insufficiency focus on its impact on protein synthesis and degradation, there is also a growing body of evidence to suggest that vitamin D supplementation in deficient individuals improves measures of mitochondrial density and function (Sinha et al., 2013; Rana et al., 2014). These studies are supported by research in rodent models, where vitamin D supplementation in deficient animals improves the balance between muscle protein synthesis and degradation, as well as measures of mitochondrial density and function (Gogulothu et al., 2020).

Due to evidence implicating compromised mitochondrial health as a key factor in the pathology of vitamin D deficiency-induced atrophy, mechanistic action of vitamin D on skeletal muscle atrophy – particularly regarding mitochondrial health – has gained attention in recent studies. Overexpression of VDR in rat skeletal muscle results in increased skeletal muscle hypertrophy, driven by increases in anabolic signaling, ribosomal biogenesis, and protein synthesis (Bass et al., 2020). While the effect of VDR overexpression on mitochondrial dynamics has not been studied, recent studies have confirmed that vitamin D regulates oxidative capacity through binding of 1,25(OH) D to the VDR in skeletal muscle (Ashcroft et al., 2020). Furthermore, mitochondrial ATP production is significantly reduced in VDR-knock down C2C12 myoblasts, supporting the idea that the absence of 1,25(OH)D signaling at the VDR may significantly reduce ATP availability. This could have important consequences for muscle regeneration, which has shown to be hampered by loss of mitochondrial capacity (Jash and Adhya, 2012). Interestingly, reductions in ATP generation following VDR knock-down occurred independently of changes in many measures of mitochondrial machinery, including electron transport system (ETS) subunits I-V, citrate synthase, and cytochrome *c* oxidase (Ashcroft et al., 2020). These results were recapitulated by an *in vivo* experiment in vitamin D deficient mice, which showed decrements in maximum oxidative capacity in the absence of differences in protein expression of ETS complexes I-V (Ashcroft et al., 2020).

Studies of VDR ablation suggest that 1,25(OH)D regulation of oxidative capacity can occur independently of significant changes in mitochondrial density and ETS protein abundance. This may be explained in part by a study showing that VDR-knockdown in C2C12 myotubes results in an increase in optic atrophy 1 (OPA1) abundance, which was proposed to be a compensatory mechanism to rescue deficits in mitochondrial function resulting from vitamin D deficiency (Ashcroft et al., 2020). Optic atrophy 1 is a marker of fusion of the inner membrane on mitochondria, which results in larger mitochondria and increased oxidative capacity (Kushnareva et al., 2013). In contrast, an increase in OPA1 expression was observed after vitamin D supplementation in vitamin D deficient mice with statin-induced myopathy (Ren et al., 2020), as well as in human skeletal muscle cells treated with 1,25(OH)D (Ryan et al., 2016). While these studies all suggest that 1,25(OH)D treatment or VDR expression alter OPA1, it is unclear why both VDR knockdown and 1,25(OH)D treatment led to increased OPA1 expression. Additionally, few studies have explored the effect of different vitamin D analogs on oxidative capacity, but in human skeletal muscle cells, only 1,25(OH) administration increased oxygen consumption rate, while exposure to both 25(OH)D and vitamin D_3_ significantly reduced it (Ryan et al., 2016). Given these disparate findings, future research should clarify the impact of VDR expression and different vitamin D analogs and doses on mitochondrial dynamics.

In addition to reductions in mitochondrial ATP production, another mechanism by which vitamin D deficiency may contribute to muscle atrophy is excess mitochondrial ROS production (Ricca et al., 2018). While normal levels of ROS are important for skeletal muscle signaling following injury, excessive ROS production that overwhelms protective antioxidant systems can be deleterious to muscle health (Le Moal et al., 2017). Vitamin D deficiency has been shown to increase lipid (Cielen et al., 2016; Dzik et al., 2018) and protein (Bhat and Ismail, 2015; Dzik et al., 2018) oxidation in skeletal muscle. Additionally, vitamin D deficiency causes alterations in antioxidant enzyme activities (Bhat and Ismail, 2015; Dzik et al., 2018). Interestingly, while vitamin D deficiency was associated with increased superoxide dismutase (SOD) activity in humans experiencing chronic lower back pain (Dzik et al., 2018), a study of rats showed a decrease in SOD activity in the plantaris of deficient animals (Bhat and Ismail, 2015). However, both studies report that deficiency results in an increase in muscle glutathione peroxidase (GPx) activity. The difference between these two studies may result from species differences (rat vs. human), the presence of muscle pathology, or any number of other factors. Notwithstanding, vitamin D supplementation was associated with correction of alterations in SOD and GPx activities in both studies. There are clear opportunities for future studies to elucidate the impact of vitamin D status on antioxidant systems in human participants.

In several models, providing vitamin D analogs exerts a protective effect on skeletal muscle and cells undergoing oxidative stress. Administration of 1,25(OH)D *in vitro* resulted in a reduction in ROS production, lipid and protein oxidation, protein ubiquitination, muscle proteolysis, intracellular damage and gene markers for atrophy, and an increase in SOD activity and markers of mitochondrial biogenesis (Bhat and Ismail, 2015; Chang, 2019). There is a paucity of data regarding the protective effects of vitamin D analogs *in vivo*. However, one study of patients experiencing chronic lower back pain revealed that vitamin D_3_ supplementation resulted in lower Cu/Zn SOD and GPx activity in paraspinal muscle compared to deficient individuals. These differences in antioxidant activity were mirrored by lower protein and lipid peroxidation. On the other hand, the same measures in un-supplemented, vitamin D replete patients did not differ from deficient patients (Dzik et al., 2018). These results not only underscore the importance of vitamin D signaling for optimal redox balance, but also highlight how the provision of vitamin D analogs promote muscle mitochondrial health during oxidative stress (Figure 1).

## Satellite Cell Mitochondrial Function and Vitamin D

Adequate oxidative capacity is critical for skeletal muscle regeneration following injury. In addition to providing energy for protein synthesis, mitochondria play an important role in regulating SC activity. Quiescent SCs have less mitochondria and lower oxidative capacity than activated, differentiating SCs, and mitochondrial activity is a key process underlying SC activation (Latil et al., 2012). This lower oxidative capacity has been recapitulated *in vivo*, where reduced mitochondrial respiration of SCs was associated with a greater proportion of SCs expressing self-renewal markers in endurance-trained mice (Abreu and Kowaltowski, 2020). In addition to the importance of metabolic reprogramming, other mitochondrial processes are implicated in optimal SC function. For instance, competent mitophagy has been shown to be necessary for normal SC activity in a study that examined Parkin null mice (Esteca et al., 2020). Parkin is an E3 ubiquitin ligase that is essential for regulation of mitophagy. Parkin knockout resulted in increased SC proliferation and impaired differentiation in mouse skeletal muscle following injury with cardiotoxin. These changes in SC activity were accompanied by delayed skeletal muscle fiber repair and smaller muscle fibers during regeneration (Esteca et al., 2020).

Another means by which mitochondria influence SC activity is through production of ROS, which stimulate symmetric division, followed by terminal differentiation (Mohammad et al., 2019). Interestingly, a study of human skeletal muscle myoblasts showed that vitamin D treatment resulted in inhibition of myoblast proliferation that was accompanied by an increase in differentiation and mitochondrial oxygen consumption rate. The authors proposed that decreased proliferation induced by vitamin D may serve to maintain SC quiescence and thus maintain the stem cell population in muscle. Conversely, the increase in mitochondrial oxygen consumption rate likely serves to power the increase in synthesis of metabolic machinery that accompanies differentiation and mature myotube formation (Montenegro et al., 2019). Taken together, these data suggest that vitamin D may modulate SC activity *via* alterations in mitochondrial density or function. Since measures of mitochondrial density and ROS production were not assessed, it is difficult to speculate on the nature of the changes in mitochondria that caused increased oxygen consumption rate, and whether alterations in ROS production may have contributed to the changes in SC activity. More research is needed to determine how vitamin D signaling affects mitochondrial function and regulation of SC activity.

Taken together, the global effect of vitamin D on mitochondrial function and the presence of VDR in SCs of regenerating muscle (Srikuea et al., 2012) suggest an interplay between mitochondria and SCs during muscle repair. Indeed, CYP27B1 is expressed in mitochondria, suggesting an intrinsic link between vitamin D activation and mitochondria (Nakamura et al., 1997). However, more research is needed to fully understand the effects of vitamin D on the relationship between mitochondria and SC activity following skeletal muscle injury. Elucidation of the precise nature of signaling between the VDR, SCs, and mitochondria may provide avenues for treatment of skeletal muscle atrophy where one or more of these components in compromised.

## Vitamin D Analogs and Skeletal Muscle Injury

A potential regenerative role of the vitamin D system in injured skeletal muscle is supported by improved cellular turnover and enhanced muscle function with subcutaneous administration of vitamin D_3_ after crush injury in rats (Stratos et al., 2013). With vitamin D_3_ treatment initiated immediately after injury, proliferation of cells in the interstitium of injured skeletal muscle is elevated with a concurrent decline in necrotic cells (Stratos et al., 2013) – supportive of enhanced activity of mononuclear cells with potential roles in muscle repair, such as various immune cells, macrophages, and fibrogenic cells. Though vitamin D_3_ administration enhanced activity of mononuclear cells within the injured skeletal muscle, Pax7+ SC abundance and muscle morphology were not clearly altered at any time point through 42 days post-injury. At the same time, vitamin D_3_-exposed rats showed higher peak tetanic torque after the severe crush injury when compared with vehicle controls (Stratos et al., 2013). Interestingly, the timing, delivery, dose, and vitamin D analog may be crucial to optimize muscle regeneration after injury. When 1,25(OH)D (the bioactive form of vitamin D) is provided in a delayed manner by intramuscular injection 4 days after BaCl_2_ muscle injury, no regenerative benefit is observed (Srikuea and Hirunsai, 2016). Both physiological and supraphysiological delayed intramuscular administration of 1,25(OH)D elevated VDR protein expression in injured muscle, but neither delayed dose produced larger muscle fibers 8 days after injury. In fact, mice receiving a supraphysiological delayed dose of 1,25(OH)D showed impaired SC differentiation and subsequent *de novo* myogenesis and presented with injured skeletal muscle having smaller muscle fibers and excessive accumulation of fibrotic materials (Srikuea and Hirunsai, 2016). In both the aforementioned studies where different vitamin D analogs were provided with different timing, routes of administration, and doses after muscular injury, muscle morphology was not positively altered toward an enhanced regenerative phenotype. This is despite improved muscle function with immediate systemic delivery of vitamin D analogs after injury (Stratos et al., 2013; Srikuea and Hirunsai, 2016). It’s worth noting that rodents consumed standard diets formulated to provide sufficient vitamin D. Therefore, it’s possible that vitamin D_3_ or 1,25(OH)D administration would affect deficient animals differently. *In vivo* protocols including both deficient and replete animals would help determine the means by which treatment with different vitamin D analogs and dosing affect muscle recovery and function after injury.

Vitamin D_3_ supplementation in young males with insufficient serum 25(OH)D concentrations resulted in improved knee extensor torque output 2 and 7 days after a damaging bout of exercise (Owens et al., 2015) suggestive of enhanced muscle regeneration to support superior muscle function. Here, insufficiency for recruitment purposes was defined as 25(OH)D < 75 nmol/L, above the Institutes of Medicine (IOM) cut point of 50 nmol/L. This study included men in both insufficiency and sufficiency ranges as defined by the IOM, which is common in human studies investigating vitamin D status and muscle phenotypes (Table 1). Nonetheless, in skeletal muscle-derived myoblasts isolated and cultured from these same young men, administration of 1,25(OH)D enhanced differentiation and myotube fusion and resulted in larger myotubes after an *in vitro* mechanical wound injury (Owens et al., 2015). These data suggest that supplementing vitamin D_3_ in young people with marginally insufficient vitamin D status may optimize regenerative processes in skeletal muscle after muscular injury. This is particularly relevant given the high prevalence of vitamin D deficiency in athletes (Farrokhyar et al., 2015) – associated with a higher prevalence of muscle strain injuries (Rebolledo et al., 2018) – and the general population across the lifespan (Holick, 2017).

## Discussion

Within the last decade, it has become clear the vitamin D receptor is expressed in skeletal muscle and has integral roles in both recovery following injury and maintaining mitochondrial function. What remains to be determined are the precise mechanisms by which it exerts these actions and how varying forms, doses, and timing of vitamin D analogs affect the outcomes. Though mounting evidence indicates that vitamin D_3_ supplementation supports mitochondrial health and oxidative capacity while reducing oxidative stress in deficient patients, it is unknown what circulating concentration of vitamin D promotes “optimal” mitochondrial health, especially in the context of injuries causing oxidative damage where it is established that some vitamins turn over more quickly (Kesavan et al., 2003; Barbosa et al., 2009). Moreover, different studies evaluating strength-associated outcomes have had overlapping sufficiency and insufficiency definitions and equivocal results, which makes interpretation exceedingly difficult. Though different countries and professional bodies recommend different cut points, future studies require consistent 25(OH)D cut points for analysis of the efficacy of specified outcomes and should evaluate the effect of dosing and timing. Specifically, it would be helpful to compare directly efficacy of a single megadose and sustained daily supplementation after muscle injuries. Though supplementation prior to injury may be realistic in some high-risk settings, it would also be helpful to establish how soon after injury doses should be administered since prior supplementation will not always be feasible.

Different natural vitamin D analogs and doses have been used to elucidate vitamin D’s cellular functions (Table 2). Most *in vitro* studies administer the active vitamin D metabolite 1,25(OH)D, which is a hormone regulating about 3% of the genome. Though valuable mechanistic insight may be gained from this approach, concentrations of this hormone vary 10,000 fold across studies applying treatments to C2C12 myoblasts and human skeletal-derived muscle myoblasts (Ryan et al., 2016; Montenegro et al., 2019). Few *in vitro* studies include treatments with 25(OH)D, but it is the vitamin D metabolite that skeletal muscle cells would be exposed to *in vivo*. Moreover, it is established that 1,25(OH)D alters mineral metabolism and carries the risk of clinically dangerous hypercalcemia, so it is typically prescribed for patients with hyperparathyroidism, chemotherapies enhanced by calcitriol, and conditions promoting hypocalcemia (Quarles et al., 1988; Muindi et al., 2002; Tartaglia et al., 2005). Animal studies we reviewed utilized oral and injectable forms of vitamin D_3_, 25(OH)D, 1,25(OH)D; however, no study compared more than one analog in the same experiment. Inclusion of both 25(OH)D and synthetic vitamin D analogs that do not target vitamin D’s classical mineral-regulating pathways (Brown and Slatopolsky, 2008) in future *in vitro* and *in vivo* studies would add considerably to the literature.

**Table 2:** Forms and doses of vitamin D analogs vary significantly across studies.

Author	Form	Dose	Model	Outcome
Bhat and Ismail, 2015	1,25(OH)D	1 or 10 nM	C2C12 myotubes	Reduced oxidative stress and proteolysis
Chang, 2019	1,25(OH)D	1, 10, or 100 nM	C2C12 myotubes	Reduced oxidative stress
Montenegro et al., 2019	1,25(OH)D	100 nM	Human skeletal muscle-derived myoblasts	Inhibited proliferation, increased differentiation and oxygen consumption
Owens et al., 2015	1,25(OH)D	10 or 100 nM	Human skeletal muscle-derived myoblasts	Improved muscle cell migration dynamics
Ryan et al., 2016	1,25(OH)D	0.01, 0.1, or 1 nM	Primary human skeletal muscle cells	Only 1,25(OH)D increased oxygen consumption
	25(OH)D	1 nM		
	Vitamin D_3_	1 nM		
Srikuea et al., 2012	1,25(OH)D	20 nM	C2C12 myoblasts and myotubes	1,25(OH)D and 25(OH)D inhibited myoblast proliferation
	25(OH)D	2,000 nM		
Chogtu et al., 2020	25(OH)D	0.1 μg/kg/day oral	Wistar rats 6–8 weeks old (unknown sex)	Attenuated statin-induced increases in plasma creatine kinase
Keskin et al., 2018	Vitamin D_3_	8.3 mg/kg subcutaneous injection	Male Wistar rats, 8–12 weeks old	Increased nitric oxide levels in muscle following ischemia/reperfusion injury
Ren et al., 2020	Vitamin D_3_	1,000 IU/kg/day oral	Male C57 BL/6 mice, 10–12 weeks	Reduced statin-induced myopathy and improved mitochondrial cristae shape
Srikuea and Hirunsai, 2016	1,25(OH)D	1 μg/kg TA muscle wet weight or 1 μg/kg mouse body weight intramuscular injection	Male C57BL/6 mice, 10 weeks	High dose decreased satellite cell differentiation, delayed regenerative muscle fiber formation, and increased muscular fibrosis
Stratos et al., 2013	Vitamin D_3_	8.3 mg/kg body weight subcutaneous injection	Male Wistar rats, unknown age	Increased muscle cell proliferation after crash injury and did not alter VDR expression

Furthermore, sport and muscle research has suffered from limited inclusion of female subjects and animals. The vast majority of animal studies we reviewed included only male rodents, and prior evidence indicates that vitamin D metabolism is affected by sex (Dam et al., 2009; Heidari and Haji Mirghassemi, 2012). Though the inclusion of both biological sexes creates variability within an experiment, future studies should include both sexes or, particularly for *in vivo* studies, include only female animals when funding and power analyses support the inclusion of a single-sex.

In summary, *in vitro* and *in vivo* models of 1,25(OH) exposure, VDR overexpression and knockdown, as well as supplementation in deficient rodents and humans, clearly indicate a role for vitamin D in the regeneration of muscle and supporting mitochondrial health. Deeper mechanistic inquiries are needed to illuminate how differing vitamin D analogs, timing, and initial vitamin D status affect skeletal muscle regeneration and mitochondrial function and how these processes may be inherently linked.

## Author Contributions

CL, CB, AK, BM, NT, AZ, and JF wrote the manuscript. CL, CF, and JF created the tables and figures. CL, CB, AK, BM, NT, AZ, CF, and JF edited the manuscript. All authors contributed to the article and approved the submitted version.

### Conflict of Interest

The authors declare that the research was conducted in the absence of any commercial or financial relationships that could be construed as a potential conflict of interest.
